# Characterization of Thrombin Generation Curve Shape in Presence of Platelets from Acute Venous Thromboembolism Patients

**DOI:** 10.3390/jcm9092892

**Published:** 2020-09-07

**Authors:** Jeremy Lagrange, Bianca Wagner, Markus Nagler, Vincent ten Cate, Alejandro Pallares Robles, Thomas Koeck, Steffen Rapp, Jürgen H. Prochaska, Henri M. Spronk, Philip Wenzel, Wolfram Ruf, Hugo ten Cate, Philipp S. Wild, Marina Panova-Noeva

**Affiliations:** 1 Center for Thrombosis and Hemostasis (CTH), University Medical Center of the Johannes Gutenberg-University Mainz, 55131 Mainz, Germany; jeremy.lagrange@inserm.fr (J.L.); Vincent.tenCate@unimedizin-mainz.de (V.t.C.); Juergen.Prochaska@unimedizin-mainz.de (J.H.P.); wenzelp@uni-mainz.de (P.W.); ruf@uni-mainz.de (W.R.); Philipp.Wild@unimedizin-mainz.de (P.S.W.); 2 Université de Lorraine, INSERM U1116, DCAC (Acute and Chronic Cardiovascular Deficiency), 54500 Nancy, France; 3 Preventive Cardiology and Preventive Medicine, Center for Cardiology, University Medical Center of the Johannes Gutenberg-University Mainz, 55131 Mainz, Germany; Bianca.Wagner@unimedizin-mainz.de (B.W.); Markus.Nagler@unimedizin-mainz.de (M.N.); Alejandro.PallaresRobles@unimedizin-mainz.de (A.P.R.); Thomas.Koeck@unimedizin-mainz.de (T.K.); Steffen.Rapp@unimedizin-mainz.de (S.R.); 4 DZHK (German Center for Cardiovascular Research), Partner Site Rhine Main, University Medical Center Mainz, 55131 Mainz, Germany; 5 Laboratory for Clinical Thrombosis and Hemostasis, Department of Internal Medicine, Cardiovascular Research Institute Maastricht (CARIM), Maastricht University Medical Center, 6200 MD Maastricht, The Netherlands; henri.spronk@maastrichtuniversity.nl (H.M.S.); h.tencate@maastrichtuniversity.nl (H.t.C.); 6 Center for Cardiology—Cardiology I, University Medical Center of the Johannes Gutenberg-University Mainz, 55131 Mainz, Germany

**Keywords:** thrombin generation, venous thromboembolism, platelets, factor Xa inhibitor

## Abstract

**Background.** Anticoagulant therapy, the cornerstone treatment in acute venous thromboembolism (VTE), strongly impacts thrombin generation (TG). Until now, the appearance of the TG curve in platelet rich plasma (PRP) from patients with acute VTE has not been investigated. **Methods.** We analyzed the shape of TG curves measured in PARP of 180 acute VTE patients. **Results.** Normal shape of TG curves was observed in 110 patients, 50 patients showed no TG and 20 patients showed biphasic TG curve. The linear regression analysis, adjusted for age, sex, VTE clinical phenotypes and therapy showed that the appearance of biphasic curves is significantly associated with female sex, presence of cancer and therapy with Factor Xa inhibitors. **Conclusions.** This study demonstrated that despite taking anticoagulants, TG in presence of platelets is still present in the majority of acute VTE patients. Appearance of unusual TG curves is strongly related to the intake of anti-Factor Xa inhibitors. The clinical relevance of biphasic TG curve appearance requires further investigation.

## 1. Introduction

Thrombin is the key protease of the coagulation system with both procoagulant and anticoagulant functions. This protease regulates the activity of the coagulation cascade, and plays an essential role in activating platelets, critical for cell-dependent thrombin amplification [1,2]. Thrombin generation (TG) assays, measuring the total amount of thrombin formed in time, could provide important information on thrombotic as well as bleeding tendencies [3]. The calibrated automated thrombogram (CAT) is an in vitro technique that has been predominantly applied in platelet poor plasma (PPP), platelet free plasma (PFP) and platelet-rich plasma (PRP).

The CAT assay has emerged as an important method addressing the overall potential of a plasma sample to form thrombin, and a promising diagnostic tool for hypo- and hypercoagulability phenotyping [4]. Higher TG potential assessed in platelet poor plasma (PPP) has been correlated with incident venous thromboembolism (VTE) and VTE recurrence [5,6]. In acute VTE patients, it remains challenging to assess the TG phenotype, as anticoagulant therapy dramatically impairs TG. On the other hand, the modification of TG in the presence of platelets of VTE patients taking anticoagulants has not been yet addressed. Furthermore, the introduction of direct oral anticoagulants (DOACs) in the management of VTE patients has constituted another unknown variable for TG evaluation. The presence of factor Xa inhibitor (rivaroxaban) in plasma has been shown to alter the appearance of the TG curve with formation of a biphasic peak (or camel-back shaped) curve instead of a normal curve [7].

The interplay between the coagulation factors and platelets is pivotal in hemostasis regulation, however it has been poorly explored in the general clinical setting. Data from a population-based study showed that TG assessed in PRP in subjects at risk for a cardiovascular disease (CVD) presented with higher peak height and ETP compared to those not at risk for CVD. In addition, platelet-dependent TG correlated to traditional cardiovascular risk factors, particularly with obesity [8]. More recently, in an in vitro experimental setting, we explored the determinants of TG curve shapes in the presence of platelets and rivaroxaban. The study showed that TG in platelet rich plasma (PRP) from healthy donors spiked with rivaroxaban developed in biphasic TG curves as a result, from a dissociation between the amplification and thrombin-dependent propagation phase [9]. In the present study, we aimed to investigate the appearance of TG curves in PRP from acute VTE patients enrolled in the Genotyping and Molecular Phenotyping in Venous Thromboembolism project (GMP-VTE). We further aimed to understand the clinical determinants of the biphasic shape TG curve by using a multivariable logistic regression model, accounting for all potential confounders.

## 2. Experimental Section

TG measurement in PRP was assessed in a subgroup of 180 individuals with confirmed VTE, randomly selected from the GMP-VTE project (N = 693 full cohort), as described in detail before [10]. GMP-VTE project included subjects with confirmed VTE from the parent studies, the VTEval and FOCUS BioSeq project, two prospective observational cohort studies with comprehensive biobanking, from Germany [11,12]. All participants gave written informed consent before entering the study. The study has been conducted in accordance with the declaration of Helsinki.

Blood drawing was performed at the inclusion of the subjects in the study when patients presented with signs and symptoms of an acute VTE. Citrate blood samples were transported by hand to the Platelet Epidemiology laboratory and centrifuged at 200× *g* for 10 min at room temperature to isolate PRP. TG was performed by the calibrated automated thrombogram (CAT, Thrombinoscope BV, Maastricht, The Netherlands) assay in freshly isolated PRP (with adjustment for platelet concentration to 150,000 platelets/µl using autologous PPP), as previously reported [8]. In the absence of corn trypsin inhibitor use, TG was triggered with 1 pM tissue factor (TF) to minimize potential contact activation, as recommended [13]. More precisely, 20 μL PRP-reagent (1 pM TF) were added to 80 μL PRP. After 10 min pre-warming at 37 °C in the fluorometer, the reaction was started by adding 20 μL of a low affinity fluorogenic substrate for thrombin (Z-Gly-Gly-ArgAMC) and calcium chloride mixture (FluCa). To correct for inner filter effects and substrate consumption, TG measurements were calibrated against a signal from the calibration well obtained in a sample from the same plasma (80 μL PRP), supplemented with a fixed amount of thrombin—α2-macroglobulin complex (20 μL of thrombin calibrator) and 20 μL of FluCa by means of Thrombinoscope software (Thrombinoscope BV, Maastricht, The Netherlands). All CAT reagents were purchased from Stago Deutschland GmbH (Düsseldorf, Germany).

All clinical and laboratory data underwent quality control by a central data management team. Two observers checked independently the curves and then compared the ranking of the curves’ evaluation. Data were reviewed for completeness by predefined algorithms and plausibility criteria. Differences between groups for normally distributed data, reported as mean (standard deviation), were tested using the t-test. Categorical variables, presented as absolute numbers and percentages, were tested using the Wilcoxon test. Multivariable logistic regression analysis was used to assess the association between biphasic shape curve, as dependent variable, and age, sex and clinical characteristics, including VTE phenotype, cancer and anticoagulant therapy as independent variables. Because of the explorative character of the analysis, a significance threshold was not defined for *p*-values, and these should be interpreted as a continuous measure of statistical evidence. Statistical analysis was performed with software program R, version 3·6·1 (Lucent Technologies, Murray Hill, USA) (http://www.R-project.org).

## 3. Results and Discussion

Table 1 shows the clinical characteristics between subjects included in the present subsample and remaining individuals from the large GMP-VTE project. No important differences were observed between the present VTE subsample and the remaining GMP-VTE sample, in terms of age, sex, presence of CVRFs, comorbidities, factor Xa inhibitor use and hormonal contraceptive therapy. The subsample showed negligible differences regarding platelet count, heparin and antiplatelet agents use and VTE phenotype compared to the remaining GMP-VTE sample.

The clinical characteristics of VTE patients, included in our subsample, according to TG curve appearance are presented in Table 2.

**Figure 1:**
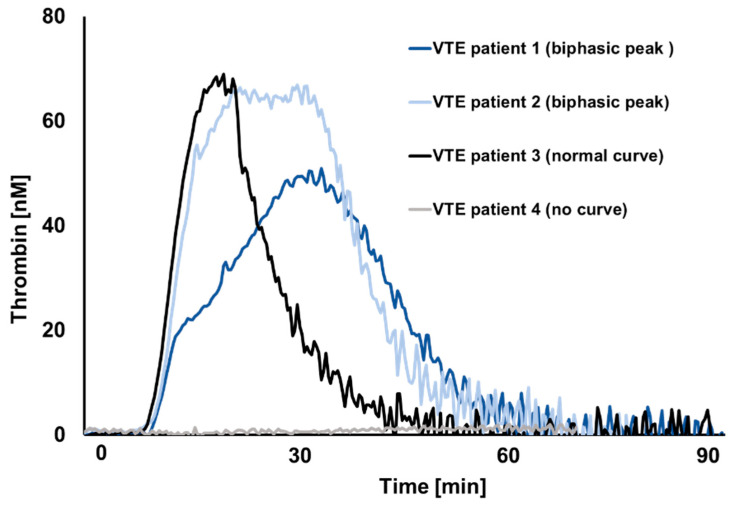
Representative thrombin generation curves from acute VTE setting.

A normal shape of the TG curve was observed in 110 patients, biphasic curves in 20 patients, and in 50 patients, no TG was observed (flat TG curve), as shown in the Figure 1. Patients with biphasic TG curve were more often females, and more frequently had cancer compared to patients with normal shape curve. Regarding the VTE phenotype, the proportion of individuals with isolated deep vein thrombosis (DVT) was lowest (5.0%) in the group of biphasic curve compared to the group with no curves (10.0%) and normal curves (29.1%). The combined pulmonary embolism (PE) plus DVT phenotype was highest in the no curves group (69%). Negligible differences between the groups were observed for the use of heparin, whereas FXa inhibitors were more frequently reported by subjects with a biphasic shape of the TG curve as compared to the no curves and normal curves groups (50% vs. 20.0% vs. 20%), respectively. No important differences between the groups were observed regarding the use of antiplatelet agents and hormonal contraceptive therapy. The results on TG in PRP showed lower peak height and velocity in subjects presenting with biphasic TG curve, compared to subjects with normal TG curve. Lag time and endogenous thrombin potential (ETP) were no different between these two groups.

The logistic regression analysis, adjusted for age, sex, VTE clinical phenotypes and FXa inhibitor, confirmed that biphasic curves were relevantly associated with female sex, cancer and intake of FXa inhibitors (Table 3). Further adjustments for traditional cardiovascular risk factors and heparin use showed no change in the observed associations.

The appearance of a biphasic curve has been shown to result from changes in TG kinetics, predominantly TG velocity calculated with a formula including TG peak height and lag time [9]. Indeed, our results showed lower peak height and velocity in patients presenting with biphasic curves compared to those showing a normal bell-shaped TG curve. Factor Xa inhibitors were strongly related to the appearance of biphasic curves. It has been reported that FXa inhibitors show greater effect on the maximum concentration of thrombin formed (peak height) than on ETP [9]. Our study confirms that ETP was not different between the two groups of non-flat TG curve shapes [9,14]. Moreover, FXa inhibitors were shown to act more on decreasing the speed of TG than completely abolishing the TG [9]. In addition, lag time was shown to be a reliable parameter that correlates with minor bleeding risk following FXa inhibition (with rivaroxaban), which is not the case with vitamin K antagonist [15]. Synergistic effects in reducing platelet activation and platelet-dependent TG have been demonstrated for rivaroxaban with single or dual antiplatelet agents [16]. The results of the COMPASS trial (Cardiovascular Outcomes for Peoples Using Anticoagulant Strategies) that included patients with stable atherosclerotic disease further confirmed that the combination of both rivaroxaban and aspirin in patients with atherosclerotic vascular disease generated a superior cardiovascular outcome [17]. However, major bleeding events occurred in 3.1% (rivaroxaban plus aspirin patients) vs. 1.9% (patients under aspirin), stressing the importance of assessing the interaction between coagulation and platelets in order to improve risk stratification and limit the occurrence of adverse events. Our study showed that female sex is strongly associated with the appearance of the biphasic curves. It has been reported that sex can differently affect the bleeding risk in patients on rivaroxaban used for thromboprophylaxis [18]. Cancer was also identified as an important determinant of biphasic TG curves. Low molecular weight heparin use in cancer-associated thrombosis was shown not significantly to affect the amount of TG after one month therapy, suggesting the persistence of a residual prothrombotic state [19]. The sustained TG in cancer patients with an acute VTE is at least in part due to enhanced platelet activation and release of procoagulant extracellular vesicles into the circulation [20,21]. Among 21 cancer patients with acute VTE, 7 individuals reported hematological malignancy and 14 individuals reported solid cancer from different organ systems. This heterogeneity prevented further analysis according to cancer type.

## 4. Conclusions

We recently presented evidence that biphasic TG curves in the presence of rivaroxaban result from a protraction of TG duration and dissociation of amplification phase and thrombin-dependent propagation phase.

Our present study demonstrated that, in the majority of acute VTE patients, TG in presence of platelets is still available for interpretation, despite taking anticoagulants. Presence of abnormal TG curve was strongly related to the presence of anti-FXa therapy.

Further studies are needed to assess the clinical relevance for the presence of biphasic TG, for the monitoring of anticoagulant treatment and clinical outcome of acute VTE patients.

## Figures and Tables

**Table 1:** Comparison of study subjects’ characteristics between VTE subsample and the remaining Genotyping and Molecular Phenotyping in Venous Thromboembolism project (GMP-VTE) sample.

	GMP-VTE	VTE Subsample	*p*-Value
Number (N)	513	180	-
Sex (females), % (N)	44.8 (230)	40.0 (72)	0.29
Age (years), mean (SD)	60.3 (15.9)	60.9 (16.0)	0.63
BMI (kg/m²), median (IQR)	28.3 (24.7/32.0)	28.3 (24.5/31.9)	0.85
Platelet count (10^9^/L), median (IQR)	239.0 (189.7/297.3)	221.5 (178.0/268.6)	0.01
Leukocytes (10^3^/L), median (IQR)	8.50 (6.53/11.30)	8.48 (6.82/11.10)	0.83
**Cardiovascular risk factors**			
Arterial hypertension, % (N)	49.5 (249)	55.8 (91)	0.18
Diabetes mellitus, % (N)	12.4 (62)	15.9 (26)	0.29
Smoking, % (N)	16.9 (82)	17.9 (29)	0.81
Obesity, % (N)	35.5 (172)	36.5 (65)	0.85
**Comorbidities**			
Atrial fibrillation, % (N)	4.2 (21)	5.5 (9)	0.52
CHF, % (N)	4.5 (22)	6.1 (10)	0.40
CAD, % (N)	7.1 (35)	8.5 (14)	0.61
Stroke, % (N)	5.0 (25)	5.5 (9)	0.84
PAD, % (N)	3.8 (12)	6.1 (8)	0.32
Cancer, % (N)	12.5 (63)	11.8% (21)	0.89
**VTE phenotype**			
Isolated PE, % (N)	18.3 (86)	21.1 (37)	0.43
Isolated DVT, % (N)	30.4 (156)	21.1 (38)	0.02
PE+DVT, % (N)	48.6 (229)	57.1 (100)	0.06
**Therapy**			
Heparin, % (N)	57.0 (292)	67.8 (122)	0.01
FXa inhibitor, % (N)	26.0 (133)	23.3 (10)	0.06
Anticoagulants *, % (N)	75.6 (387)	80.6 (145)	0.18
Antiplatelets **	23.2 (119)	33.9 (61)	0.01
Contraceptives, % (N)	4.1 (21)	6.7 (12)	0.22

N, number; %, Percentages, based on non-missing information; SD, standard deviation; IQR, interquartile range; BMI, body mass index; CHF, chronic heart failure; CID, chronic inflammatory disease; CKD, chronic kidney disease; CLD, chronic liver disease; CAD, coronary artery disease; PAH, pulmonary arterial hypertension; PAD, peripheral artery disease; VTE, venous thromboembolism; PE, pulmonary embolism; DVT, deep vein thrombosis; includes agents with the following Anatomical Therapeutic Chemical codes: * B01AA, B01AB, and B01AF. ** Clopidogrel and acetylsalicylic acid.

**Table 2:** Study subjects’ characteristics.

	Normal Curve	Biphasic Curve	*p*-Value (Biphasic vs. Normal)	No Curve	*p*-Value (No Curve vs. Normal)
Number (N)	110	20	-	50	-
Sex (females), % (N)	33.6 (37)	55.0 (11)	0.081	48.0 (24)	0.11
Age (years), mean (SD)	59.0 (16.7)	63.5 (13.7)	0.19	64.3 (14.7)	0.046
BMI (kg/m²), mean (SD)	28.4 (5.2)	30.7 (7.8)	0.23	29.6 (7.0)	0.31
Platelet count (10^9^/L), median (IQR)	222.0 (176.2/285.8)	232.5 (189.7/293.6)	0.18	219.0 (178.0/250.0)	0.90
Leukocytes (10^3^/L), median (IQR)	8.52 (6.83/11.08)	9.15 (7.82/10.72)	0.35	7.65 (6.59/11.27)	0.66
**Cardiovascular risk factors**					
Arterial hypertension, % (N)	49.5 (49)	58.8 (10)	0.60	68.1 (32)	0.049
Diabetes mellitus, % (N)	13.9 (14)	17.6 (3)	0.71	19.6 (9)	0.46
Smoking, % (N)	23.0 (23)	0 (0)	0.023	13.3 (6)	0.26
Obesity, % (N)	35.5 (39)	36.8 (7)	1.00	38.8 (19)	0.72
**Comorbidities**					
Atrial fibrillation, % (N)	8.0 (8)	0 (0)	0.60	2.1 (1)	0.27
CHF, % (N)	6.0 (6)	5.9 (1)	1.00	6.5 (3)	1.00
CAD, % (N)	6.9 (7)	5.9 (1)	1.00	12.8 (6)	0.35
Stroke, % (N)	3.0 (3)	0 (0)	1.00	12.8 (6)	0.029
PAD, % (N)	4.8 (4)	8.3 (1)	0.50	8.3 (3)	0.43
Cancer, % (N)	9.3 (10)	25.0 (5)	0.059	12.0 (6)	0.58
**VTE phenotype**					
Isolated PE, % (N)	19.4 (21)	33.3 (6)	0.22	20.4 (10)	1.00
Isolated DVT, % (N)	29.1 (32)	5.0 (1)	0.024	10.0 (5)	0.0083
PE+DVT, % (N)	50.9 (55)	61.1 (11)	0.46	69.4 (34)	0.037
**Therapy**					
Heparin, % (N)	71.8 (79)	45.0 (9)	0.035	68.0 (34)	0.71
FXa inhibitor, % (N)	20.0 (22)	50.0 (10)	0.0089	20.0 (10)	1.00
Anticoagulants *, % (N)	80.9 (89)	75.0 (15)	0.55	82.0 (41)	1.00
Antiplatelets #, % (N)	59.1 (65)	45.0 (9)	0.33	58.0 (29)	1.00
Contraceptives, % (N)	6.4 (7)	10.0 (2)	0.63	2.0 (1)	0.44
**TG in PRP**					
Lag time (min)	11.67 (8.07/18.89)	12.78 (10.76/18.11)	0.59	n.a.	-
Peak Height (nM)	63.30 (33.39/102.76)	43.08 (32.14/66.90)	0.094	n.a.	-
ETP (nM.min)	1323.27 (926.37/1691.17)	1464.06 (1194.51/1775.80)	0.38	n.a.	-
Velocity (nM/min)	5.21 (1.83/11.80)	1.82 (1.46/3.45)	0.0025	n.a.	-

N, number; %, Percentages, based on non-missing information; SD, standard deviation; BMI, body mass index; CHF, chronic heart failure; CID, chronic inflammatory disease; CKD, chronic kidney disease; CLD, chronic liver disease; CAD, coronary artery disease; PAH, pulmonary arterial hypertension; PAD, peripheral artery disease; VTE, venous thromboembolism; PE, pulmonary embolism; DVT, deep vein thrombosis; TG, thrombin generation; PRP, platelet rich plasma; ETP; endogenous thrombin potential; n.a., non-available (the thrombin generation curve was a flat line); includes agents with the following Anatomical Therapeutic Chemical codes: * B01AA, B01AB, and B01AF; # Clopidogrel and acetylsalicylic acid.

**Table 3:** Clinical determinants of biphasic TG curves.

	OR (95% CI)	*p*-Value	OR * (95% CI)	*p*-Value
Sex (females)	5.14 (1.48; 17.88)	0.010	7.36 (1.76; 30.83)	0.0063
Age (10 years)	1.21 (0.81; 1.80)	0.35	1.20 (0.73; 1.96)	0.47
Cancer	6.06 (1.33; 27.70)	0.020	6.14 (1.04; 36.25)	0.045
Isolated DVT	0.19 (0.02; 1.77)	0.15	0.19 (0.02; 1.94)	0.16
Isolated PE	1.61 (0.43; 5.95)	0.48	1.25 (0.24; 6.61)	0.79
FXa inhibitor	7.60 (2.07; 27.93)	0.0023	5.87 (1.33; 25.98)	0.020

* Adjusted additionally for arterial hypertension, diabetes mellitus, obesity, and heparin. TG, thrombin generation; OR, odds ratio; CI, confidence interval; PE, pulmonary embolism; DVT, deep vein thrombosis; FXa, Factor Xa.
